# Transcriptomic analyses reveal the expression and regulation of genes associated with resistance to early leaf spot in peanut

**DOI:** 10.1186/s13104-020-05225-9

**Published:** 2020-08-11

**Authors:** Limin Gong, Suoyi Han, Mei Yuan, Xingli Ma, Austin Hagan, Guohao He

**Affiliations:** 1 Tuskegee University, Tuskegee, AL 36088 USA; 2 Henan Academy of Agricultural Sciences, Zhengzhou, 450002 China; 3 Shandong Peanut Research Institute, Qingdao, 266000 China; 4 Henan Agricultural University, Zhengzhou, 450002 China; 5 Auburn University, Auburn, AL 36830 USA

**Keywords:** Defense response, Differentially expressed gene, Gene regulation, RNA-seq, Transcriptome

## Abstract

**Objective:**

Early leaf spot (ELS) caused by *Cercospora arachidicola* (Hori) is a serious foliar disease in peanut worldwide, which causes considerable reduction of yield. Identification of resistance genes is important for both conventional and molecular breeding. Few resistance genes have been identified and the mechanism of defense responses to this pathogen remains unknown.

**Results:**

We detected several genes involved in disease resistance to ELS through transcriptome analysis. Using RNA-seq technology, one hundred thirty-three differentially expressed genes (DEGs) were identified between resistant and susceptible lines. Among these DEGs, coiled coil-nucleotide binding-leucine rich repeat (NLR) type resistance genes were identified as duplicated R genes on the chromosome B2. Peanut phytoalexin deficient 4 (PAD4) regulator of effector-triggered immunity mediated by NLR resistance proteins and polyphenol oxidase (PPO) genes play important roles in early leaf spot resistance. Our study provides the useful information on plant response to *C. arachidicola* infection in peanut. The results suggest that a few major genes and several factors mediate the resistance to ELS disease, showing the characteristics of quantitative trait in defense responses.

## Introduction

Peanut (*Arachis hypogaea* L.), originated in South America, is a major oilseed crop and is cultivated in tropical and subtropical regions of the world. Peanut production around the world totaled ~ 38 million tons in 2015 [1]. However, peanut productivity is severely constrained by several biotic and abiotic stresses. Amongst those stresses, early leaf spot (ELS) caused by *Cercospora arachidicola* Hori and late leaf spot (LLS) caused by *Cercosporidium personatum* (Berk & Curtis) are serious foliar diseases in peanut worldwide. Peanut yield losses may reach up to 50% worldwide due to these two diseases [2] and their management is highly dependent on multiple fungicide applications. The best strategy to avoid the significant economic loss is to develop resistant cultivars.

The genetic nature of resistance to early and late leaf spot diseases is quantitative and inherited independently [3, 4]. Resistance to ELS is associated with resistance components, such as longer latent period, reduced sporulation, smaller lesion diameter, and lower infection frequencies [5]. Dominant gene action controls resistance components and epistasis played a major role in the inheritance of resistance components [6]. Sequencing of the genome of *C. arachidicola* provides relevant information for the advancement of resistant cultivars and is a useful resource to aid in the selection of target resistance genes for enhanced disease control [7].

RNA sequencing (RNA-seq) is an effective technology for understanding of metabolic processes, genome-wide quantification of gene expressions, and identification of key genes associated with the traits of interest. Recently, RNA-seq analysis provides insights into transcriptome profiles associated with several traits in peanut. Transcriptomes have revealed the mechanism of salinity resistance [8, 9], nodulation [10], pod development [11, 12], aflatoxin resistance [13, 14], defense related genes to *Ralstonia solanacearum* [15], and resistance to LLS [16] in peanut. However, transcriptome profiling analysis of resistance to ELS has been limited. To explore the mechanism of resistance to ELS in peanut, the transcriptional profiles of two sister inbred lines, resistant (R) and susceptible (S) lines to ELS, from a F_9_ RIL population were investigated under *C. arachidicola* infection. The objectives of this study were to (1) compare the differentially expressed genes (DEGs) between R and S lines, (2) understand the possible roles of DEGs in plant defense response, (3) identify resistance genes to ELS disease.

## Main text

### Materials and methods

#### Plant materials and inoculation

Two peanut lines 904 and 1006 selected from a F_9_ RIL population [17] derived from a cross of Florida-07 × GP-NC WS 16 were used in this study. The parent GP-NC WS 16 is resistant and Florida-07 is susceptible to ELS, respectively. Early leaf spot disease was evaluated in the E.V. Smith Research Center, Tallassee, Auburn University (2016 and 2017). The inbred line 904 was selected due to its higher resistance level similar to the resistant parent and the sister line1006 demonstrated higher susceptibility to ELS compared to the other lines in the F_9_ RIL population. Nine seeds of each line were individually planted in 40 Magenta GA-7 plant culture boxes containing a potting mix.

#### Fungus culture and inoculation

A total volume of 0.5 ml *C. arachidicola* conidial suspension was applied to the foliage of each plant by spraying. The inoculated plants were kept at room temperature with 100% humidity in the closed culture box. Four leaves from each plant were collected at three time points, T1 (4 h), T2 (24 h), and T3 (44 h) after inoculation, with three biological replicates. RNA isolation from 18 leaf samples (2 lines × 3 time points × 3 replicates), library construction and RNA sequencing were performed in the Beijing Genomics Institute (BGI).

### Identification of differentially expressed genes (DEGs), hierarchical clustering analysis, gene ontology (GO) enrichment analysis, and KEGG enrichment analysis

The differentially expressed genes (DEGs) were identified using EBSeq algorithms [18] based on the gene expression level with cutoff: fold change ≥ 2.00 and posterior probability of being equivalent expression (PPEE) ≤ 0.05. DEGs were compared between lines and within a line at different time points by Heatmap plot and Hierarchical clustering analysis. Gene ontology (GO) and KEGG annotation were performed using phyper, a function of R, with pvalue calculating formula in hypergenometric test (resource/hypergenometric.jpg), then calculate false discovery rate (FDR) for each p-value. The FDR < 0.01 was defined as significant enriched.

## Results

### RNA-seq and transcriptome profiles between R and S lines

To compare gene expression of plants infected by the *C. arachidicola*, the resistant and susceptible lines were used and RNA sequencing of 18 samples, including samples from the R line and the S line at T1, T2, T3 time points with three biological replicates, were used for transcriptome analysis. All clean reads were subjected to genome mapping. On average, 83.42% reads were mapped to the reference genome and 66.48% reads of each sample were mapped to only one location of the reference genome. The uniformity of the mapping results for each sample suggested that the samples were comparable.

### Identification of differentially expressed genes (DEGs)

**Fig. 1:**
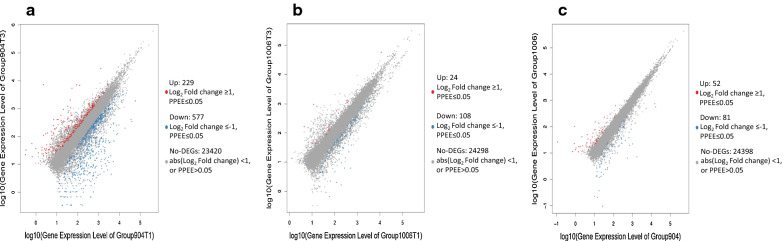
Comparison of DEGs after inoculation. Scatter plot **a** T1 (4 h) vs T3 (44 h) in the line 904 (R); **b** T1 vs T3 in the 1006 line (S); **c** lines 904 (R) vs 1006 (S)

A total of 1711 DEGs were detected from all three time points between two lines. Among these DEGs, 595 were up-regulated and 1116 were down-regulated. The resultant data showed that the number of down-regulated DEGs was significantly higher than those of up-regulated DEGs in both lines (Additional file 1: Figure S1). Due to gene expression dynamically changing between T1 (4 h) and T3 (44 h), the comparison of DEGs focused on T1 vs T3. When comparing R and S lines at T3, 133 DEGs including 52 up and 81 down-regulated were identified according to gene expression level with fold change ≥ 2 (Additional file 2: Table S1). The summary of DEGs was illustrated in the Fig. 1.

### Gene ontology and KEGG enrichment analysis of DEGs

Gene ontology (GO) enrichment analysis was performed to analyze the functions of DEGs. Metabolic process with 137 and 25 genes were dominant in the biological process category in R and S lines, respectively. Catalytic activity (181 genes) and binding (102 genes) were significant active responses in the molecular function category in the R line, whereas only 33 and 25 genes with the same function in the S line, respectively, indicating there was a higher number of expressed genes in R line than in S line between T1 and T3 (Additional file 3: Figure S2).

**Fig. 2:**
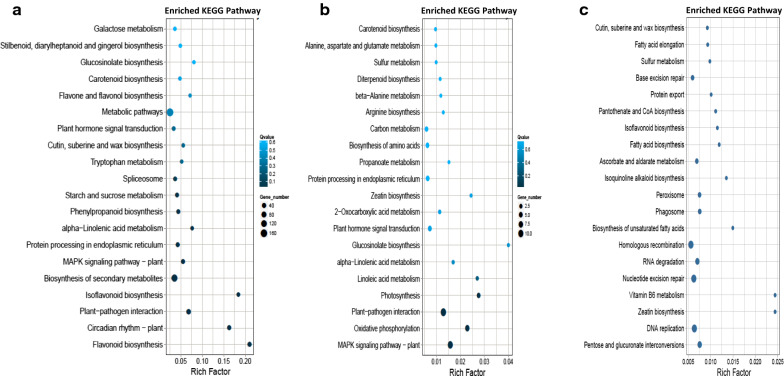
Comparisons of enriched KEGG pathway of DEGs. **a** T1 (4 h) vs T3 (44 h) in 904 R line, **b** T1 vs T3 in 1006 S line, and (**c**) 906 R vs 1006 S line. Dot size and color refer to gene number and the corrected *P* value, respectively

To further explore biological functions and gene interaction, Kyoto Encyclopedia of Genes and Genomes (KEGG) enrichment analysis was used. For the R line, comparison of DEGs with T1 vs T3 showed 11 KEGG pathways were significantly enriched, including biosynthesis of secondary metabolites, phenylpropanoid biosynthesis, MAPK signaling pathway-plant, flavonoid biosynthesis, plant-pathogen interaction, circadian rhythm-plant, isoflavonoid biosynthesis, etc. While in the S line, 4 KEGG pathways including photosynthesis, plant-pathogen interaction, oxidative phosphorylation, MAPK signaling pathway-plant were significantly enriched for DEGs between T1 and T3 (Fig. 2).

### DEGs related the resistance to ELS

There were 133 DEGs with 52 up and 81 down-regulated genes identified between R and S lines (Additional file 2: Table S1). Some DEGs were crucially involved in plant responses to the pathogen infection. For instance, nine of such genes were significantly up-regulated in the S line while down-regulated in the R line based on the log2 fold change, including two nucleotide binding-leucine rich repeats (NLRs), Interleukin-1 receptor-associated kinase 4, ATP-dependent RNA helicase, Nuclear pore complex protein Nup85, and E3 ubiquitin-protein ligase. On the other hand, 10 different DEGs including phytoalexin deficient 4 (PAD4), Interleukin-1 receptor-associated kinase 4, ATP-dependent RNA helicase, ATP-binding cassette, Serine/threonine-protein kinase, Pectinesterase, Auxin responsive GH3 gene family, and Polyphenol oxidase were significantly up-regulated in the R line but down-regulated in the S line (Table 1). The sequence of putative NLRs were showed in Additional file 4: Table S2.

**Table 1:** Key DEGs related with the resistance to early leaf spot

Gene ID	Length	904 line expression^a^	1006 line expression^a^	Log2 fold change	PPEE	Up/down	Kegg orthology
Araip.IU5QT	612	33.7176	5.4789	− 2.5519	0.0272	Down	Phytoalexin deficient 4 (PAD4)
Araip.JWB3B	1659	16899.7185	571.6533	− 4.8849	4.33E−06	Down	Interleukin-1 receptor-associated kinase 4
Araip.56E0J	816	1003.7531	22.1262	− 5.4828	4.09E−08	Down	Interleukin-1 receptor-associated kinase 4
Araip.IK389	2844	321.6808	132.3308	− 1.2793	3.66E−15	Down	ATP-dependent RNA helicase DHX36
Araip.JZ9DY	1208	23.2084	1.8579	− 3.4296	5.59E−09	Down	ATP-binding cassette, subfamily A
Araip.Z7XRR	1629	34.2064	7.2808	− 2.1825	6.23E−12	Down	ATP-dependent RNA helicase DHX36
Araip.K2RZJ	820	255.9979	37.3803	− 2.7651	1.96E−12	Down	Serine/threonine-protein kinase ULK4
Araip.V8NJN	1773	272.7235	33.1558	− 3.0277	0.0206	Down	Auxin responsive GH3 gene family
Araip.XC3V5	1413	665.8651	61.8233	− 3.4221	0.0046	Down	Pectinesterase
Araip.4G0SR	1758	542.5663	6.3732	− 6.3405	2.18E−11	Down	Polyphenol oxidase
Araip.U0YME	2421	26.8468	79.3904	1.5527	0.0003	Up	CC-NBS-LRR
Araip.JMY44	2472	8.9355	24.9109	1.4462	0.0018	Up	CC-NBS-LRR
Araip.498BF	438	6.3517	31.0765	2.2334	0.0002	Up	Interleukin-1 receptor-associated kinase 4
Araip.BV0ZS	1467	17.1560	136.8627	2.9722	0.0413	Up	Interleukin-1 receptor-associated kinase 4
Araip.CR951	2301	0.7055	11.2789	3.4915	1.67E−08	Up	ATP-dependent DNA helicase PIF1
Araip.X0YWA	795	41.0504	139.8412	1.7602	1.73E−09	Up	ATP-dependent RNA helicase DBP3 (DEAD-box)
Araip.DV5FU	390	17.1582	35.0932	1.0185	0.0006	Up	Nuclear pore complex protein Nup85
Araip.XEA6C	339	1.4038	11.1285	2.7271	4.39E−07	Up	Nuclear pore complex protein Nup85
Araip.ZN9XB	240	6.4672	24.2974	1.8578	0.0421	Up	E3 ubiquitin-protein ligase RNF38/44

^a^Line 904 is resistant and line 1006 is susceptible to early leaf spot

## Discussion

The development of disease-resistant plants is important in mitigating the losses of productions. Understanding the mechanism of plant-pathogen interactions and searching for resistant resources is a prerequisite in plant breeding. Plant responses to pathogen attack are involved in several defense strategies against pathogens. The first barrier for pathogen invasion is the uses of plant reprogramming the cell wall damaged by virulent pathogens, resulting in pathogen-associated molecular pattern (PAMP)-triggered immunity (PTI) [19, 20]. This basal resistance layer is controlled by lipase-like protein phytoalexin deficient 4 (PAD4) either in combination with enhanced disease susceptibility 1 (EDS1) or independent of EDS1 [21, 22]. PAD4 is required for activation of responses during some other gene-for-gene resistance responses [23] and plays the potential role of defense signaling genes in quantitative disease resistance [22]. Several reports have demonstrated the increasing resistance against pathogens via expressing PAD4, such as in Arabidopsis [24]. In this study, PAD4 was significantly down-regulated in the S line compared to the R line, suggesting that the lower expression level of PAD4 might affect the resistance to ELS in the susceptible genotype.

Because PAD4 is an important component of the downstream signaling in a different immune pathway, the reduced expression of PAD4 may also have a negative effect on resistance pathways. Several other genes participated in various pathways responding to ELS pathogen challenges were identified in this study. These genes encoded various enzymes to conspire in biological processes. For instance, expression of pectinesterase gene was significantly reduced in the S line that could result in weak cell walls due to degradation of pectin, a critical cell wall component. In plants, pectinesterase plays a role in plant response to pathogen attack by influencing numerous physiological processes [25]. However, the repressed expression of pectinesterase gene led to degrade cell wall integrity, thus increase *C. arachidicola* colonization of leaf tissue and enhance disease development. [26] described that the impact of pectin methyl esterification on plant-pathogen interactions and on the dynamic role of its alteration during pathogenesis. If these passive defenses were breached, the plant innate immune system would launch active defenses [27]. Nevertheless, due to the down-regulation of PAD4 in the S line, it lost the functions of the signaling accumulation for downstream activities.

Besides PAD4, polyphenol oxidase (PPO) genes also significantly down-regulated in the S line. Normally, PPO is up-regulated by abiotic and biotic stresses though the responses to stresses varies within PPO gene families by plant species [28]. Overexpression of PPO displayed enhanced resistance to pathogen in wheat, chickpea, tomato, and populus [29–33]. Conversely, down-regulation of PPO resulted in increased disease susceptibility. For instance, silencing of PPOs leads to increased susceptibility to disease in tomato [34] and to insects in tomato and cotton [35, 36]. [37] studied the interactions of host with fungi in pearl millet and reported that the resistant genotypes showed high, rapid accumulation of localized levels of PPO while susceptible genotypes failed to accumulate PPO even after consideration time. In this study, significant down-regulation of PPO in the S line could enhance susceptibility to ELS disease.

In addition to PTI, another type of defense response is activated by resistance proteins as sensors that indirectly or directly recognize specific effectors from many different pathogens, leading to effector-triggered immunity (ETI). The resistance genes, NLR with N-terminal Toll-interleukin-1-receptor domain or coiled coil domain encoding resistance proteins, are a major class of resistance genes that evolved to intercept these effectors. Two putative NLRs were identified as DEGs between the R line and S line in this study. Two R genes possessed the same DNA sequences with several SNPs and were closely located in the B2 chromosome. Higher R gene expression at the earlier reaction period in the susceptible genotype implied that resistance genes trigged by pathogen infection responded quicker in the susceptible than the resistant genotype. [16] observed the same scenario in a different peanut population responding to LLS disease. The susceptible genotype was eventually failed in defense response might be due to the lower expression of PAD4. Overexpression of PAD4 would allow us to have further insight into the plant response in the susceptible genotype.

## Limitations

The resistance genes identified from differentially expressed genes still need to validate by functional study, such as loss of function mutation targeting on these genes.

## Supplementary information

**Additional file 1: Figure S1.** Summary of DEGs among different groups. X-axis represents comparison of DEGs between each group and Y-axis refers to the number of DEG. Red color indicates up-regulated DEGs and blue color for down-regulated DEGs.

**Additional file 2: Table S1.** List of 133 differentially expressed genes identified between the R line and the S line.

**Additional file 3: Figure S2.** Gene ontology (GO) enrichment analysis showed DEGs involved in three function categories. A. T1 (4 h) vs T3 (44 h) in R line; B. T1 vs T3 in S line; C. R vs S lines.

**Additional file 4: Table S2.** DNA sequences of resistance genes to early leaf spot disease in peanut.

## Data Availability

All raw fastq sequences for these eighteen libraries were deposited in the National Center of Biotechnology Information (NCBI). The BioProject number is PRJNA494675 (https://www.ncbi.nlm.nih.gov/sra/PRJNA494675).

## Abbreviations

DEG: Differentially expressed genes
FDR: False discovery rate
FPKM: Fragments per kilobase of exon per million mapped reads
GO: Gene ontology
KEGG: Kyoto encyclopedia of genes and genomes
MYB: Myeloblastosis
PAD4: Phytoalexin deficient 4
PDA: Potato dextrose agar
PPEE: Posterior probability of being equivalent expression
PPO: Polyphenol oxidase
ROS: Reactive oxygen species
