# Supplementary figures and images for: Transcriptomic analyses reveal the expression and regulation of genes associated with resistance to early leaf spot in peanut
Source: BMC Res Notes. 2020 Aug 11;13:381. doi: 10.1186/s13104-020-05225-9 (PMC7418390; doi:10.1186/s13104-020-05225-9)

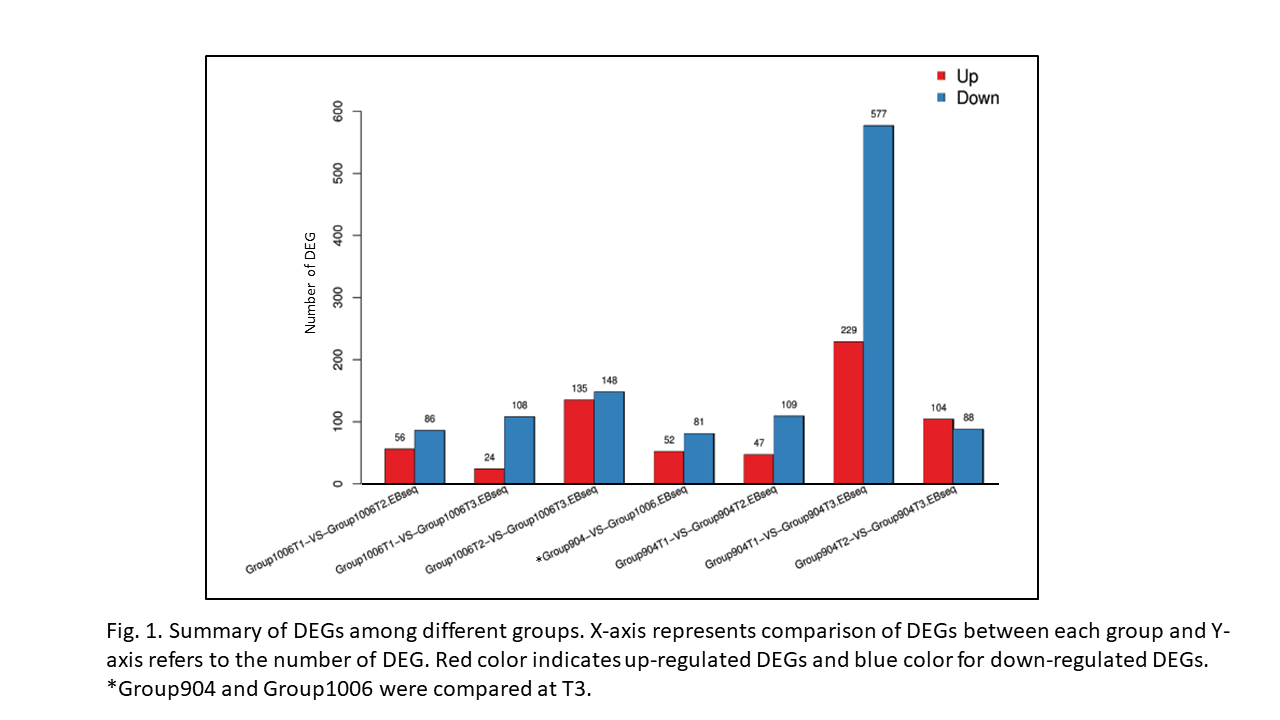

Supplement: Supplementary file 1 — Additional file 1: Figure S1. Summary of DEGs among different groups. X-axis represents comparison of DEGs between each group and Y-axis refers to the number of DEG. Red color indicates up-regulated DEGs and blue color for down-regulated DEGs. [file 13104_2020_5225_MOESM1_ESM.tif]
