# Supplementary material for: Transcriptomic analyses reveal the expression and regulation of genes associated with resistance to early leaf spot in peanut
Source: BMC Res Notes. 2020 Aug 11;13:381. doi: 10.1186/s13104-020-05225-9 (PMC7418390; doi:10.1186/s13104-020-05225-9)
Supplement: Supplementary file 3 — Additional file 3: Figure S2. Gene ontology (GO) enrichment analysis showed DEGs involved in three function categories. A. T1 (4 h) vs T3 (44 h) in R line; B. T1 vs T3 in S line; C. R vs S lines. [file 13104_2020_5225_MOESM3_ESM.pdf]

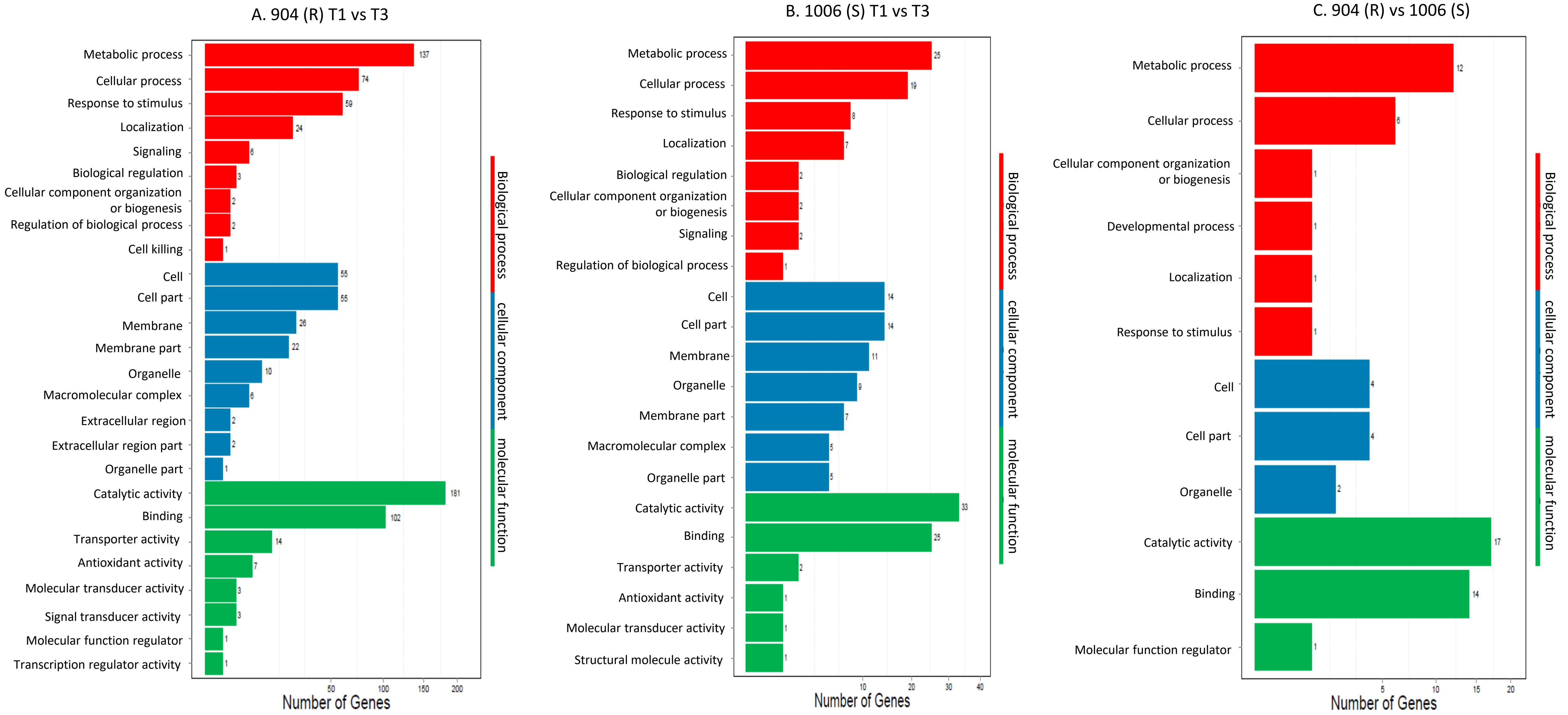

Fig. 4. Gene ontology (GO) enrichment analysis showed DEGs involved in three function categories. A. T1 (4 h) vs T3 (44 h) in R line; B. T1 vs T3 in S line; C. R vs S lines.
