# Supplementary material for: Transcriptomic analyses reveal the expression and regulation of genes associated with resistance to early leaf spot in peanut
Source: BMC Res Notes. 2020 Aug 11;13:381. doi: 10.1186/s13104-020-05225-9 (PMC7418390; doi:10.1186/s13104-020-05225-9)
Supplement: Supplementary file 4 — Additional file 4: Table S2. DNA sequences of resistance genes to early leaf spot disease in peanut. [file 13104_2020_5225_MOESM4_ESM.docx]

Table S2: DNA sequences of resistance genes to early leaf spot disease in peanut.

>Araip.B08:126723005..126723971 (PAD4)

TCATTTTCCAATGCCATTCTCAAAGAAAGATGGGGTGCCAACTTCATTCACATGGTAACAAAGCATGACATCATGCCAAGGATTCTCTTTGCACCTACAATGCCTCACATTGCTCAACTCAATTCCTTGCTTCAATTTTGGCAATTTTCTATGGCTAATCCAAGCTCTCTTGGGAACCTTGCGATGCAAGTAACTGACGGGGACAAAGCTGAGTTGTTCAGCTTTGTCACGACTTACTTGCATCATGCAGCTACCCAAGAGGGAGTCGAAGGGTTCTTTCGTCCCTTTGGGAGCTTCCTTTTTGTTTCGGACGAAGGAGCAGTGTGTGTGGAGAGTTCTGCCGCAGTTATAAAGATGATGCACTTGATGTTTGTAACAAGTTCTCCAGATTCTAGTATTGAAGATCATCTAAAGTATGGAGATTATGTCAATAAATTGTCATTGCAATTCTTGAGCAAGAAATTGTCATTGCAAGGGATTGTTCCTAATTGTAGCTATGAAGCAGGGCTTGAATTGGCACTCCACTCTTCAGGGATAACAAGACAGGTAAAAAACATTTTTTCCTTTCATTGATAGTTTGCATTGGAAGAAAGTGGTTTAACTGAACAATTACCTTTTTTTCAACTGATAGCAACCGGCTTTCGAGCCGGCCAAGGAGTGCCTTAAGGAGGCTAGGAGAGCAGGTCCATCGCCGGCGCTGAAGGCTGCTCGGCTTGCAATTACCTTATCCAAATTTGTACCTTACAGGGCAGAAATAGAATGTTACAAAGCTTGGTGTGATCAACAGAGTGATCAAATGGGATACTATGATTTGTTCAAGAGGAGAGGCTCTTCAAAAAGGGAAATGAAGGTCAACATGAACCGTATCAAGCTTGCAAGGTTCTGGAACAATGTGGTTGACATGCTGGAGAGGAATGAGCTGCCTTATGACTTTGAGTTGCGAGCAAAATGGATCAACGCTTCGCAT

>Araip.B02:4108921..4111605 (CC-NB-LRR)

ATGGCTGAAGTTGTTTCTGGTGTGGCATCAACACTCTTAGGCAATTTAGCAACAAAATCTTTTCAAGAGATTGCTCTGGCATGCGGTCTTAAAGATGATGTAAAAAAGTTTGAAAGTTCTTTGAGAACCATCAAAGCATATCTCATAGATGCTGAGAACAAGCAAGCAAAAAACCGCAGTATAGATGAGTGGTTGAAGCAACTCAGAGAGGCATTTGATGATGCTGGTGACATATTAGATGAAATAGAGTATGAAGCAAAACTTAATGAAGTGGTCAAAATGTATGGAAGCATTAGCACGAAGGTTCGCCGATTCTTCTCATACACAAGTAATCCACTTGTATTTCGCATCAAAATGGCCCACAAAATCAAAGATATGAAACAGAAGATGGATGAAAAAATAAGAGAAGGGAGAGACTTGGGTATAGTTGAACAACATGTCAACACTCCAGCTCTGGAGCACAATTTACCATGGCGAGAGACTGCTTCTTCATTGCCTTTCCGTGTGTGTGGTAGACTTGAAGAGAAAGAGGAGATTATAAAGTCATTGATGACACAAAAATCAGAAGCTAATAGTATTGATGTGATCTCAATTGTTGGGATTGGTGGTTTGGGAAAGACTACACTTGCACAAATGGCTTACAATGATACCCCAGTGAAGGCGAATTTCGATCCCTTAATGTGGGTGTGTGTATCTGATGATTTTGATGTCAAGAAGCTAATACAAAGAATCATTCATGCAGCCACAAAGAGAGAGAATTTCGTGGATGCAAATTTTAGTTTGGAATATATGATATCTCTTCTCAATCAAACGTTGCATGGTAAAAAATTCTTACTCGTGTTAGACGATGTTTGGAATGAAAACCACAGCAAATGGGATGAATTGAGAAATCACTTGTTAGAAGCAGGTGGTGACAAAGGCAGCAAAATTATAGTGACCACTCGTAGCAGAAAAGTTGCTGACATTATGGGGAGTAATCTTGTAATGAAATTGGAAGGACTTCCTGAGAATGAATGTTGGCGGCTCTTTGCAAAATGTGCATTCCAAGAAGAGAAGGACGAAGAGAAGTACCCAAGGCTAAAGCAAATTGGGGAGCAAATTGTTAAAAAATGCAAAGGAGTACCCTTGGCTATAACAACTTTGGGTTGCTTGCTTAGATCAAAATCACATGATGAAAATGAGTGGAGAAAAATAAGGGATAGTGAGGTGTGGAATCTCGATCAAGAAGAAACTGACATTTTGCCATCACTCAAATTGAGTTACAATCACTTGCCATCAGAAGTAAAACAATGTTTTTCATACTGCTCTTGTTTTTCAAAGGATTACGAATATGTTGCTATTGATTTGATTATGTTGTGGATGGCTCATGGACTCCTCCAACCTACACGCGAAGAGGAAGATGCAGAAGATATTGGGGAGTTGTATATTAAGAAGCTTGTTTCAGCATCTTTACTTCAAATTGAAGGAGATTCTAACCCCTACTTTGATTTCAAAAATTTAATGACATTTAAAATACTCAAAATGCATGATCTTGTACATGATCTTGCACAATTAACAATGAAAGAGTCAAGCAGGACAAGAACCGTTATGCAAGAGGGGCAACAAGAAGCATTGATAGAATGGACCTCCGACAAGTTCAACTATCTGAGGGTGTTGCACCTAACAAAATATATGGAATTGAGCTCGTTGCCTGATGATTGCTTTGCCATAATGAAGAAGCACTTGAGATATCTCTTTCTTAGAAATTGTCCTAGTTTGAAAAAGCTTCCTGATTCCATTTGTAAGCTGCAAAGTTTGCAGAGTTTGGGCCTTGGTTGTGACAGCCTTCAAGAACTTCCCAAAAACATGAACAAACTCATCTATCTACAATATTTGTTTTTGATGGGCATCAAAATTACAAGTTTGTCTTCAATGAATATAGGGCGCTTCCAACAACTCAAATGCTTGTATCTTTTGAAATGTTCAAGGTTAGTGTCCGTACCAAGTGCTGTTGGTCGCTTGACTACTTTAAAGAAGTTGGGATTTTATGAGTGTGAAGAGCTGGTGAATTTTGAAGATGAAGAGGAAGAAGGAAAGCAACATGTGGTAATAAATAATTTAAACCTTCAATTATTCTCAATCATTGGGTCTGCGAAGTTGGATGCTTTACCAAAATGGCTTGAAAGAGCTACTAAATTGCGACATTTGAGTATAATGTTAACAGGGATAAAATCATTGCCCACAAGGTTGCCGATGACCTCTCTTGAAGAACTTTATATCCTTCTATGTGCAGAATTATCATCTCTTCCTAACATGGATCAAGCTCATAATCTTCAGTATTTAGAGATATATTATTGTCCCACATTATATGCAAAGTACAATAAAGAGACAGGTCCAGATTGGCCCAAAATTGCTCATATTCCATATTGCAAGGTTAGTTCAAATAATCTATCTCATAATATTACAAATCTAACCATACTGGTTTATTATTATTATTATTATTATTATTATTATTATTATTATTATTATTATTATTATTATTATTATTATTATGCATGAAAAATTAATTGTTGATGTTTTATGTTTAATTAACCAGTTAGAGTTATATCTTACTCTCCTTATAATTAATTCTAGAACTTTTTAAATTGACAATCTAATTTTCTTTCTAAATAATTGTGTATAATTTATTTATAAAAGGTATGGTAG

>Araip.B02:3957980..3961994 (CC-NB-LRR)

ATGGCTGATGTTGTTTCTGGTGTGGCATCAACACTCTTAGGCAATCTAGCAACAAAATCTTTTCAAGAGATTGCTCTGGCATGTGGTCTTAAAGATGATGTAAAAAAGTTTGAAAGTTCTTTGAGAATCATCAAAGCATATCTCATAGATGCTGAGAACAAGCAAGCAAAAAACCACAGTATAGATGAGTGGTTGAAGCAACTCAGAGAGGCATTTGATGAAGCTGGTGACATATTAGATGAAATAGAGTATGAAGCAAAACTTAATGAAGTGGTCAAAATGTATGGAAGCATTTGCACGAAGGTCCGCCGGTTCTTCTCATATACAAGTAATCCACTTGCATTTCGCATCAGGATGGCTCACAAAATCAAAGATATGAAACAGAAGATGGATGAAAAAATAAGAGAAGGGAGAGACTTGGGTATAGTTGAACAATATGTCAACACTCCAACTCTTGAACACAATTTACCATGGCGAGAAACTGCTTCTTCATTGTCTTCCCGTGTGTGTGGTAGACTTGAAGAGAAAGAGGAGATTATAAAGTCATTGATGACACAAAAATCAGAAGCTAATAGTATTGATGTGATCTCAATTGTTGGGATCGGAGGTTTGGGAAAAACTACACTTGCACAAATGGTTTACAATGATATCTCAGTGAATGAGCATTTTGATCCCTTAATCTGGGTGTGTGTATCTGATGATTTTGATGTCAAGAAGCTAATACAAAGAATCATTCATGTGGCCTCAAAGGGAGAGAATTTCGTGGATGCAAATTCTAGTTTGGAATATATGATATCTCTTCTCAAGCGAACGTTGTGTGGTAAAAAATTCTTACTCGTGTTAGACGATGTTTGGAATGAAATCCACAGCAAATGGAATGAATTGAGAAATCACTTGTTAGGAGTAGGTGGTGACAAAGGCAGCAAAATTATAGTGACCACTCGTAGCAGAAATGTTGTTGACATTGTGGGGAGTAATCTTGTAATAAAATTAGAAGGACTTCCTGAGAATGAATGTTGGAGGCTGTTTACAAAATGTGCATTCCAAGAAGAGAAGGAAGAAGAGAAGTACCCAAGGCTAAAGCAAATTGGGGAGCAAATTGTTAAAAAATGCAAAGGGGTGCCCTTGGCTATAACAACTTTGGGTTGCTTGCTTAGATCAAAATCCCATGATGAAAATGAATGGAGAAAAATAAGGGATAGTGAGGTGTGGAATCTCAATCAAGAAGAAAGTGACATTTTGCCATCACTCAAATTGAGTTACAATCACTTGCCACCAGAAGTAAAGCAATGTTTTTCATACTGCTCTTGTTTTCCAAAGGATTATGAATATGATGGTATTGAGTTGATTATGTTCTGGATGGCTCATGGACTCCTCCAACCTTCACGCGAAGAGGAAGATGCAGAAGATATTGGAGAGTTATATCTTAAAAAGCTTGTTTCAACATCTTTACTTCAAATTGATGAAGTATATTCTTTCTTGTTTCCCAAGATTGGAAATTTGATGGCATTTAAAAATCTCAAAATGCATGATCTTGTACATGATCTTGCAAAATTAACAATGAAAGAGTCAAGCAGAACAAGAACCGTTGTGCAAGAGGGGCAACAAGAAGCATCAATAGAATGGACCTCCGAGAAGTTCAACTATCTGAGGGTGTTGCACCTAACAAAAGATATGGAGTTGAGCTCGTTTCCTGATGATTGCTTTGCCACAATGAAGAAGCACCTGAGATATCTCTTTCTTGGAAATTGTCCTAGTTTGAAAAAGCTTCCTGATTCCATTTGTAAGCTTCAAAGTTTGCAGAGTTTGGGCCTTCATTGTGACAGCCTTCAAGAACTTCCCAAAAACATGAACAAACTCATCTATCTACAATATTTGCTTTTGATGGGAATCAAAATTACAAGTTTGTCTTCAATGAATATAGGGCGCTTCCAACAACTCAAATTCTTGTATCTTTTCAATTGTTCAAGGTTAGTGTCCGTACCAAGTGCTGTTGGTCGCTTGACTACTTTAAAGAAGCTGGTCTTTCTTTGGTGTGAAGAGCTGGTGAATTTTGAGGATGAGGAGGAAGAAGGAAAGCAACATGTGCAAGTAAATAATTTAAACCTTCAATTATTCTCAATCGCTGGATCAAAAAAGTTGAATGCTTTACCAAAATGGCTTGAAAGAGCTACTAAATTGCAATATTTGAGTATAAGCATAACAGGGATAAAATCATTGCCCACAAGGTTGTCGATGACCTCTCTTGAACAACTTTATATCGATCGGTGTGAAGAAATGTCATCTCTTCCTAACATGGATCAAACTCATCATCTTCAATATTTAAAGATATATTATTGTCCCGAATTATATGAAAGGTACAATGAGGAGACAGGTCCAGATTGGCTCAAAATTGCTCATATCCCACAATGCGAGGTTAGTTCAAATAATCTCTCTCATAATATTACAAATCTAACCATACTGGTTTATTATTATTATTATTATTTCAATTTTTGCATGAATAATTAATTGTTGGAATTTTAATTAACCAGTTAGTGTTATATCTTACTCTCCATATAATTAATTCTAGAACTTTTTAAATTGACAATATAATTTTCTTTCTAAATAATTGGGTATAATTTATATATAAAAGGTATGGTAGAGTGAACTAATTAGTCCTTATAATATGGAATACTTCCAAATTTTGAAAATGCAATATAATATAGGTTATAAATTTATACTTTCAACTTGAGTCACACCCTAGACATGTTTCATTTTAATTTTAAGAAAATAATGATATTAAGATATTAAAATCAAAATTTTTATTAAATGAAATAAAATTCCTTCTGTTAGTCTGTTGGTAAAAATTTTTTCAAAGTTAGTTAATCATTGCTTTTAGTTATAAAGATTTTTTTTTTTTTAACTTTTCCGATTGTTCATATCACATTGGTCAAATTCTTTCTTTTGGAGGGTGCGTAAATTGAATTTTTTTAAATAATAAAAATTAAATTTTAAATTTTTACGTTATAAAAATTATGATATTATATTATAATACTATTAAAAAAAACATAAACTGATAAAAAATATACACAAACAATTATATCTAACAATAATAATAATTATTAGTAATTCATACTAGTATATATAATTTGGATTCTACGATAATTATTATTATGGTGATTATATATTGTTAAAATTAAAATTATATATGTAACACCGTAATATTCAAATCCTTATACTCGAGTCATAAGTTAATAATAATAAGGTGGTATAACTCTCAAAGGGGAATTTTAACACATAATTACAAATAAAATTGAAAGGAGTATTAATCGAGAAGCTTGAAAAGAGTAAAAATAAAATCGCGAATTCGTAGCGCTCACGTATCGACAGTGAAGAAGAAGAAAGCAGTTCTTGTCAATTGTTTGAATTTCTTTTTAATAATCTTTCTAAATAAATACATCAAATCGATTAACATTATTATGGCTGCAGCACATGTCTTGAATTTTAGTTATGAATCTTAATATTACCGCTAATAATGGTAACTCTTTCAATTACTACGTATTATGAATTATTGTATTGAAAAGCACAATAAGCTAATTATTATGTGAAATACAAAGAATGAATAACTCTTAATCATTTGAATATTGACTATACATTATTTAAAATGAGCACAAAATTTTATTATTATTGTAATTATAGGAGTTGATGCATGAATCTTTATTTTTGGATAGTGAAATACTTTTTTATTGTCAATTGACAGAAAATGGAAATTTAGAAAATTATAAAACAATTTAATTATATTTAGTATATATATGCATGGTTGAATTAAAGCTCAAGTTATTTTAATTTCATAAGCACTAGTCTGTCTAGTGGCCATGAATTATTGCTTTGTTGATAATAATAGCATTATCTTTGCCTAATGGTTGTGTTCTAATAATTGCTTTGTTTGGCAACTTAGATCATATCTTTGAAGGAGCAGATTGAGAAGATTATTGACATGAATAGGAAGTCATTGATAAAGCTTAGAAATCAAGCA
